# Circulating Extracellular Vesicles in Gynecological Tumors: Realities and Challenges

**DOI:** 10.3389/fonc.2020.565666

**Published:** 2020-10-14

**Authors:** Carolina Herrero, Miguel Abal, Laura Muinelo-Romay

**Affiliations:** ^1^Translational Medical Oncology Group (Oncomet), Health Research Institute of Santiago de Compostela (IDIS), University Hospital of Santiago de Compostela (SERGAS), Santiago de Compostela, Spain; ^2^Nasasbiotech, S.L., A Coruña, Spain; ^3^Instituto de Salud Carlos III, Centro de Investigación Biomédica en Red de Cáncer (CIBERONC), Madrid, Spain; ^4^Liquid Biopsy Analysis Unit, Translational Medical Oncology (Oncomet), Health Research Institute of Santiago de Compostela (IDIS), Santiago de Compostela, Spain

**Keywords:** ovarian cancer (OC), endometrial cancer (EC), circulating extracellular vesicles (cEVs), biomarkers, liquid biopsy

## Abstract

Although liquid biopsy can be considered a reality for the clinical management of some cancers, such as lung or colorectal cancer, it remains a promising field in gynecological tumors. In particular, circulating extracellular vesicles (cEVs) secreted by tumor cells represent a scarcely explored type of liquid biopsy in gynecological tumors. Importantly, these vesicles are responsible for key steps in tumor development and dissemination and are recognized as major players in cell-to-cell communication between the tumor and the microenvironment. However, limited work has been reported about the biologic effects and clinical value of EVs in gynecological tumors. Therefore, here we review the promising but already relatively limited data on the role of circulating EVs in promoting gynecological tumor spread and also their value as non-invasive biomarkers to improve the management of these type of tumors.

## Introduction

Precision oncology has emerged with the aim of achieving more accurate and active treatments for individual patients on the basis of the molecular characteristics of the tumor. This personalized oncology is intimately associated with the discovery of molecular biomarkers useful in predicting tumor prognosis and therapy response, and attaining accurate disease monitoring. In this context, circulating biomarkers and liquid biopsies are key elements for implementing personalized oncology as an ideal complement to tissue biopsies and radiologic analyses. The use of circulating biomarkers clearly improves the assessment of tumor spatial and temporal heterogeneity and evolution. Therefore, together with immunotherapy applications, the use of liquid biopsies to characterize tumors has marked a revolution in oncology (1).

Presently, liquid biopsy strategies are mainly based on the characterization of circulating tumor DNA (ctDNA), circulating tumor cells (CTCs), and circulating extracellular vesicles (cEVs) as sources of proteomic and genetic information. In fact, the determination of *EGFR* mutations in non-small cell lung cancer (NSCLC) through ctDNA analyses is been used to identify candidate patients for TKI based therapy (2). Besides, accumulating scientific evidence indicates the utility of ctDNA analyses to detect other clinically relevant alterations in different genes, such as *RAS, BRAF*, or *PI3KCA* in colorectal, melanoma, or breast tumors (3–5). In addition, during the past 20 years, the study of tumor cells released into the circulation, the CTC population, has provided broad information about the molecular mechanisms favoring tumor spread and dissemination (6, 7). However, their clinical use remains anecdotal, and their application is mainly focused on translational research, owing to the difficulty of their isolation and their high heterogeneity; therefore, their analysis remains a challenge in many tumor types and clinical contexts (8, 9).

**Figure 1:**
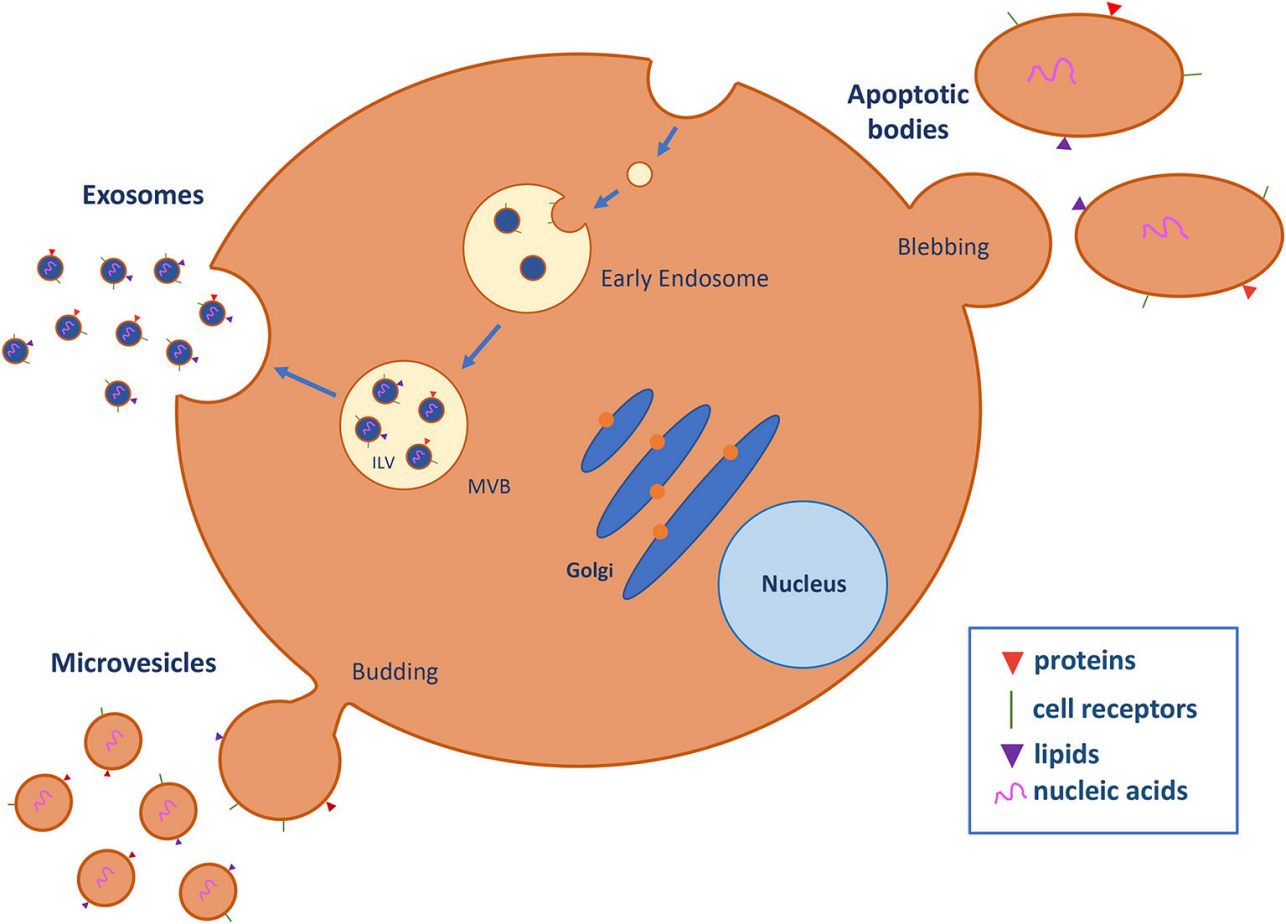
Extracellular vesicle (EV) biogenesis and secretion. EVs are classified into three major subtypes on the basis of biogenic and morphological properties: exosomes, microvesicles (MVs), and apoptotic bodies. Exosomes are nanostructures of approximately 30–100 nm in diameter that originate as intraluminal vesicles (ILV) in multivesicle endosomes (MVB), which are intermediates in the endosomal system and can fuse with the plasma membrane and secrete their contents in exosomes into the extracellular space. Microvesicles (50–2,000 nm) are generated by direct budding and fission of the plasma membrane into the extracellular space. Apoptotic bodies (500–4,000 nm) are released by the blebbing process during programmed cell death. EVs contain several cell-specific components, such as proteins, lipids, and nucleic acids (DNA, mRNA, miRNA, and lncRNA) that are transferred to target cells (10, 11).

The last of the three pillars of circulating biomarker research is circulating extracellular vesicles (cEVs), a complex population of cell-derived membranous structures secreted by numerous cell types and generated by different cellular mechanisms (described in detail in Figure 1) (10, 11). Importantly, EVs refer to three main entities: exosomes, ranging from 30 to 100 nm; microvesicles, which are large membrane vesicles of 50–2,000 nm; and apoptotic bodies, which are typically 500–4,000 nm. These structures have a pivotal role in cancer, interacting with stromal cells, favoring tumor cell growth and proliferation, and enhancing the invasiveness and metastatic ability of target cells (12). Specially, EVs are key players in the establishment of the premetastatic niches required for cancer cell dissemination and engrafting at distal sites. Premetastatic niches comprise a specialized and favorable microenvironment that facilitates colonization and promotes the survival and outgrowth of disseminated tumor cells (13). Of note, hypoxia and microenvironmental acidity are key factors influencing cell fate within the tumor microenvironment as well as the secretion of EVs (14–16), independently of tumor histology (17). These data reinforce the value of assessing EV levels as a common biomarker in cancer (18). For example, high levels of cEVs are present in the plasma of patients with glioblastoma and change over the disease course (19).

Among the cancer related mechanisms mediated by EVs, angiogenesis appears to be important for maintaining tumor growth and dissemination. In fact, EVs secreted by different tumor cells have been shown to be relevant mediators of angiogenesis (20). In addition, several studies have shown that EVs modulate drug resistance through different mechanisms. For example, HER2-positive EVs secreted by breast cancer cells bind Trastuzumab and inhibit its anti-proliferative activity (21). In addition, the release of P-glycoprotein (P-gp) via EVs has been described as another mechanism of drug resistance in breast (22) and prostate cancer patients (23). Alternative mechanisms mediated by EVs have been found to be responsible for resistance to Temozolamide in glioblastoma (24), Gefitinib in esophageal squamous cell carcinoma (25), or Tamoxifen in breast cancer (26).

In addition to the important roles of EVs in tumor promotion and their interest as therapeutic targets, as we previously described, EVs have emerged as promising cancer biomarkers because they increase in different biological fluids as a consequence of the disease (12) and have high stability in circulation, protecting their molecular cargo (proteins, mRNAs, non-coding RNAs, and single-stranded or double-stranded DNAs) from the environment. Therefore, the analysis of cEVs is a promising tool for improving the clinical management of cancer patients but is currently far from being clinically validated. For example, increased exosomal PSA levels have been shown to be a valuable biomarker for both screening and secondary prevention of prostate cancer in a clinical study (27). Rodríguez-Zorrilla et al. have shown, in a pilot study, that CAV-1 positive exosomes increase after surgery, whereas low peri-surgical levels of plasmatic exosomes correlate with better survival in patients with oral squamous cell carcinoma (28).

Other studies aiming to characterize and validate EVs as a clinical tool have focused on PC. Melo et al. have identified glypican-1-associated EVs as a potential non-invasive diagnostic tool to detect early stages of pancreatic cancer (PC) (29). Opposite, other study has described low levels of miR-let7a and high levels of exosomal miR-10b, miR-21, miR-30c, and miR-181a, differentiating better PC from healthy control and chronic disease, compared plasma CA 19-9 levels or with exosomal glypican-1 (30). Several works have demonstrated that EV miRNAs may be useful as diagnostic biomarkers in different tumor types. Ogata-Kawata et al. have identified higher levels of seven serum exosomal miRNAs (let-7a, miR-1229, miR-1246, miR-150, miR-21, miR-223, and miR-23a) in patients with primary CRC than in healthy people (31). In the same line, a recent study has described exosomal miR-19b and miR-21 as independent diagnostic factors with higher sensitivity and specificity than the routinely used clinical biomarker CEA (32). Liu et al. have shown that elevated levels of exosomal miR-23b-3p, miR-10b-5p, and miR-21-5p are independently associated with poor overall survival in patients with NSCLC (33), whereas downregulation of the exosomal miRNA let-7a-5p leads to elevated expression of the target gene BCL2L1 and poor survival of patients with lung cancer (34). More recently, a combination of miR-375, miR-655-3p, miR-548b-5p, and miR-24-2-5p has been postulated to be a good diagnostic tool to detect early breast cancer (35).

Overall, all these data evidence the major roles of EVs in cancer as an indicator of tumor complexity, and a promising diagnostic and therapeutic tool. However, as we previously highlighted, the field of EVs is now far from clinical applicability in all tumor types, particularly gynecologic cancers. Therefore, the present review aims to summarize the knowledge on the pathological functions of EVs in endometrial and ovarian cancer as well as on the clinical potential involving the analysis of their protein and miRNA cargo.

## Circulating EVs in Endometrial Cancer

Endometrial cancer (EC) is the most prevalent gynecological cancer in developed countries. Most cases are diagnosed at a localized stage with a 5-year relative survival of 95%. However, this rate decreases to 69% when regional spread exists and to 16% when distant metastasis occurs (36). Although most cases are diagnosed at early stages, 2–15% of cases develop recurrent disease, and this proportion can reach 50% in women with advanced stage EC (37). EC treatment usually consists of surgery and adjuvant radiotherapy for patients with a high risk of recurrence. Chemotherapy is administrated in patients with metastatic/recurrent disease and high-grade tumors (38), but traditional chemotherapy is less active than in other cancers because of acquired resistance and the absence of targeted therapies (39). In this regard, efforts to improve EC management have mainly focused on the discovery of new therapeutic targets, and prognostic and predictive biomarkers to better define the risk of recurrence and the response to therapy.

Several biomarkers with prognostic and predictive value have been studied in EC tissue samples, such as L1CAM, a prognostic factor for FIGO stage I tumors, and ANXA2, identified as a predictor of recurrent disease in EC (40, 41). However, none of these biomarkers have been implemented in clinical practice, because of their limited sensitivity and/or specificity. Moreover, although the potential of liquid biopsies in EC has scarcely been explored, some studies have demonstrated the feasibility of detecting both CTCs and ctDNA in blood samples from patients with different stages of the disease, mainly with high-risk or metastatic cases (42–44). These promising preclinical results support the need for exploring the clinical benefit of circulating biomarkers to stratify patients with EC and identify alternative therapies. For this purpose, cEVs present in blood, urine, or uterine aspirates constitute a valuable alternative for improving the analytical sensitivity of protein and genetic biomarkers (43). Importantly, in this regard, the eventual effects of EVs on the uterine microenvironment have been explored in the context of embryo implantation, with the transfer of the miRNA content of exosomes and microvesicles between endometrial epithelial cells and trophectodermal cells of the blastocyst (45) and in endometriosis (46), but not in the context of the endometrial carcinoma environment (47), where their role is unknown.

In searching for new clinical EV-associated biomarkers, our group has recently studied ANXA2 levels in complete plasma and the cEV fraction. We observed that ANXA2 protein is present mainly in isolated cEVs but not as a soluble protein in the plasma. Importantly, in this work, we identified higher levels of ANXA2 in cEVs isolated from plasma samples of patients with EC than healthy controls. Furthermore, the analysis of ANXA2 in plasma EVs had favorable specificity and sensitivity as an EC biomarker (AUROC = 0.74) and was correlated with tumors with high risk of recurrence and non-endometrioid histology, thus indicating the potential of cEV-based ANXA2 levels as a promising diagnostic and prognostic liquid biomarker in EC (48).

In recent years, the study of miRNAs as potential biomarkers has increased. In patients with EC, miRNAs are associated with regulation of gene expression, epigenetic dysfunction, and carcinogenesis (49). Circulating free miRNAs have also been described as potential biomarkers for early EC diagnostic and detection of tumor progression (50, 51). Of note, several studies have identified miRNAs in EVs from different body fluids. Srivastava et al. have evaluated the potential of miRNAs from urine-derived exosomes as a diagnostic biomarker for EC, showing an enrichment of miR-200c-3p in urine exosomes from patients (52). Roman-Canal et al. studied the miRNA profile in EVs from peritoneal lavage in EC. The authors found 114 miRNAs significantly altered in EVs from patients with EC. Among these, miR-383-5p, miR-10b-5p, miR-34c-3p, miR-449b-5p, miR-34c-5p, miR-200b-3p, miR-2110, and miR-34b-3p were downregulated in patients with EC and have been found to have predictive performance (AUC ~ 0.90) (53). Another study by Li et al. has explored the roles of miR-302a-enriched EVs derived from human umbilical cord mesenchymal stem cell (hUCMSC) in EC cell growth and mobility. The authors suggest that these miR-302a-loaded EVs impair cell proliferation and migration by downregulating cyclin D1 and suppressing AKT signaling, thus suggesting that EVs rich in miR-302a may be a potential therapeutic strategy in EC (54). In addition, exosomal miR-93 and miR-205 have been found to increase in serum from patients with EC, and high miR-205 levels were associated with poor tumor evolution (55). Although all these data, the application of miRNAs in EVs from EC patients is still underdeveloped, being necessary more studies to explore all their potential.

## Circulating EVs in Ovarian Cancer

Ovarian cancer (OC) is the second most frequent tumor in Western countries after cervical cancer. There are no specific symptoms or screening tests for OC. Moreover, most OCs are high grade tumors that affect one or both ovaries, and have a high dissemination capacity (56). Survival rates in patients with OC have not substantially improved in recent years, because of the lack of effective targeted therapies (57). After surgical intervention, patients with advanced OC are treated with adjuvant chemotherapy, mainly based on platinum. However, most of these cancers recur, thus representing another important challenge to improving OC survival and achieving early diagnosis of the disease (58). The most common histological subtype is high grade serous OC, which is characterized by the presence of *P53* mutations and deficiency in homologous recombination. Since the DNA repair mechanisms are often altered in high grade serous OC, they are normally highly sensitive to platinum regimens but often acquire resistance mechanisms (59). In this context, only CA-125 levels are being used as a surrogate circulating biomarker to manage the disease (60). Therefore, in OC, as with EC, there is a need for accurate non-invasive biomarkers with clinical value for improving OC diagnosis and predict patient outcomes after surgery, to facilitate more precise follow-up and therapy selection. In this sense, the study of EVs as a non-invasive liquid biopsy tool sheds light on the discovery of new diagnostic and prognostic biomarkers together with therapeutic targets for OC.

EVs optimize the microenvironment for OC colonization, promoting angiogenesis, stromal remodeling, and immunosuppression in the premetastatic niche (61). Exosomes released into ascitic fluid might result in biophysical and functional changes in fibroblasts that promote the development of a malignant tumor microenvironment in OC (62). Likewise, the crosstalk between OC cells and endothelial cells modulating tumor angiogenesis may implicate exosomes and their miRNA content (63). Exosomes released in the hypoxic tumor microenvironment may also contribute to metastasis and chemotherapy resistance in OC, thus representing an opportunity to improve treatment success (64). In fact, our group has identified roles of exosomes derived from ascites in the communication between tumor cells and their environment. We found that exosomes obtained from ascites from patients with OC promote adhesion of SKOV3 cells. We took the advantage of this finding to develop a novel tumor cell capture system (M-Trap) comprising exosomes with an adhesive capacity to capture tumor cells and to impair the generalized peritoneal spreading in OC. In a murine model of OC spread generated by intraperitoneal injection of SKOV3 cells, when the M-Trap was implanted at the peritoneum, the dissemination of SKOV3 cells became markedly different, such that the metastasis localized on the M-Trap. Thus, M-trap technology is able to remodel metastatic patterns, transforming a systemic disease into a focal disease and offering a new therapeutic approach (65).

EVs also carry several factors that suppress the immune system and are responsible for the differentiation and activation of immune suppressor cells, modulating antigen presentation, or inducing T-cell apoptosis (66). In this sense, Peng et al. have detected FasL and TRAIL in EVs isolated from ascites of patients with OC; these proteins are associated with the apoptosis of immune cells and tumor escape from the immune response (67). Another study by Czystowska-Kuzmicz et al. has identified ARG1 in OC-derived EVs as a suppressor of peripheral T-cells that promotes tumor growth and evasion of the immune system (68).

There is more literature regarding the value of EVs as biomarkers in OC than in other gynecological tumors, such as EC. Thus, different protein cargos derived from EVs have been described as potential clinical biomarkers in OC. Li et al. have observed an enrichment in Claudin 4 in plasma exosomes from patients with OC, thus indicating its value as potential biomarker in OC (69). EVs isolated from OC ascites have been also described to contain L1CAM, CD24, ADAM10, and EMMPRIN, which favor tumor progression (70). Peng et al. have identified eight proteins normally expressed in OC (CLIC4, AKT1, EMAPII, SNX3, FAM49B, FERMT3, TUBB3, and lactotransferrin) in circulating exosomes and have suggested their utility in early diagnosis (71). In addition, CD9/HER2-positive EVs were found higher in the serum from patients with OC than in healthy controls and patients with non-malignant disease (72). More recently, the presence of GSN, FGG, FGA, and LBP proteins in OC derived exosomes has been described and associated with the promotion of the coagulation dysfunction that frequently occurs in OC (73). Besides, EV proteins also have important roles in tumor staging and as biomarkers for treatment response in patients with OC. In this regard, Szajnik et al. have reported that plasma from patients with OC is characterized by higher levels of exosomal proteins than those in plasma from controls (benign disease and healthy donors), and these levels are correlated with tumor stage (74).

Beyond EV associated proteins, mRNAs or miRNAs have been also related to OC carcinogenesis and aggressiveness. Yokoi et al. have found that cEVs containing MMP1 mRNA in ascites from patients with OC induce apoptosis in mesothelial cells, thereby promoting peritoneal dissemination (75). Taylor et al. have described an EVs miRNA signature of tumor derived-exosomes that are of interest as diagnostic markers. This signature includes miR-21, miR-200a, miR-200b, miR-141, miR-200c, miR-205, miR-214, and miR-203. Furthermore, the authors have found that the exosomal miRNA profiles from the serum of patients with OC are similar and significantly distinct from the profiles observed in benign disease (76). In addition, in the serum, exosome miR-222-3p is higher in patients with OC than healthy controls, and this elevation is associated with the interactions between ovarian tumor cells and macrophages (77). Meng et al. have found that exosomal miR-373, miR-200a, miR-200b, and miR-200c are significantly higher in serum samples from patients with epithelial OC than from healthy women. Moreover, miR-200b and miR-200c are associated with poor overall survival and tumor progression (78). Xu et al. have identified an alteration in miR101 levels in tissue samples and serum exosomes from patients with OC. Their results indicate that a decrease in miR101 in serum exosomes may serve as potential diagnostic biomarker in OC (79). In addition, high levels of EV-associated miR-99a-5p have been detected in serum from patients with OC and correlated with the promotion of invasion by regulating human peritoneal mesothelial cells (HPMCs) through vitronectin and fibronectin (80). In an analysis of plasma EVs in OC and healthy controls, Zhang et al. have found that miR-106a-5p, hsa-let-7d-5p, and miR-93-5p are significantly higher in OC, whereas miR-185-5p, miR-122-5p, and miR-99b-5p are lower. The authors suggest that these differentially expressed EV-associated miRNAs may be potential diagnostic and prognostic targets for OC treatment (81). Pan et al. have also focused on the influence of exosomal miRNAs on the pathogenesis of epithelial OC. They have found significantly higher miR-21, miR-100, miR-200b, and miR-320, and lower miR-16, miR-93, miR-126, and miR-223 in exosomes from the plasma of 106 patients with epithelial OC compared with 29 healthy women. Of note, the levels of miR-200b correlate with the tumor marker CA125 and overall patient survival (82). There is also evidence of a role of EV-associated miRNAs in therapy response of OC. Kuhlmann et al. have analyzed a set of EV-associated miRNAs in plasma samples from patients with OC and different response to platinum-based regimens. This panel is differentially abundant in platinum resistant vs. platinum sensitive OC, thus suggesting their potential as biomarkers predictors for platinum resistance (83).

Other body fluids have also been explored in OC for EV associated miRNA analyses. Thus, Vaksman et al. have identified an increase in miR-21, miR-23b, and miR-29a in exosomes derived from OC effusion supernatants. These miRNAs are associated with poor progression-free survival, and high expression of miR-21 correlates with poor overall survival (84). In contrast, global miRNA characterization has shown that miR-30a-5p is up-regulated in urine samples from patients with OC compared with healthy controls. This study suggests that the increased levels of this miRNA in urine samples may be due to the secretion of exosomes from OC cells; therefore, miR-30a-5p has been proposed as a new diagnostic marker in OC (85).

All these studies show that EVs are a potential source of diagnostic and prognostic biomarkers for OC. However, many challenges must be addressed before clinical utilization of EVs in detection and treatment of OC will be possible.

## Challenges in the Clinical Application of cEVs in Gynecological Tumors

EVs play an important role in cell interaction by modulating the activity of target cells through either the action of surface proteins or trafficking with molecules between cells. This role is common among tumor types. As we previously described, EVs activity is a key element in multiple pathophysiological procedures, such as inflammatory responses, immunoregulation, carcinogenesis, tumor invasion, and metastasis. In fact, tumor-derived EVs have emerged as a new source of circulating cancer biomarkers, because they are present in all body fluids and have different molecular cargos from those of non-tumor EVs. Notably, in comparison with other circulating elements such as ctDNA or CTCs, cEVs are found in body fluids in higher concentrations, and, more importantly, they protect and stabilize their molecular cargo. Therefore, cEVs have great promise as biomarkers for different tumor types, including endometrial cancer and OC. Despite this potential, generalized implementation of EV-based biomarkers in clinical contexts remains far from reality.

One of the main challenges to improving EV applications for treatment of cancer, particularly gynecological tumors, is the limited performance of methods for the isolation and characterization of EVs. There is no technical standardization, and evidence of high specificity and sensitivity for routine clinical implementation is lacking. The need for standardized protocols includes sample collection and processing, EV isolation, and numerous strategies to analyze EV molecular cargo, thus making comparison of the results obtained in different studies difficult. In particular, EV isolation strategies are normally grouped into five isolation techniques: ultracentrifugation, polymer-based precipitation, immune-selection, density-gradient separation, and microfluidic isolation. All these strategies can be combined and applied to different body fluids. However, all these methods have some limitations. Ultracentrifugation is the most commonly used method for isolating EVs; however, it is time consuming, requires high sample volumes, and provides low recovery of EVs. An ideal method for isolation of EVs in a clinical context should enable simple use without a need for complex equipment, and should be fast and compatible with many samples. Currently, there are easily used technologies for EV isolation from liquid biopsies, which have been successfully applied in gynecological tumors; these include ExoQuick, ExoSpin, and ExoGAG (48, 84). The results obtained with these technologies are promising; however, most of the studies on endometrial and ovarian tumors have been performed in limited cohorts of patients (Table 1). Therefore, there is a clear need for large scale clinical studies using robust technologies to answer relevant questions about gynecological tumors. For OC, the key clinical need is the validation of biomarkers for early diagnostic and screening, in addition to markers of prognostic and therapeutic value. In EC, clinicians require new accurate biomarkers to stratify patients who have a higher risk of recurrence after surgery and new markers to guide therapy selection in metastatic settings. The improvement of cEV isolation technologies, together with the application of multi-omics strategies to characterize their molecular cargo, and the selection of larger and well defined cohorts of patients for studies, will be critical in the near future to enable clinical translation of circulating EVs in the management of gynecologic tumors.

**Table 1:** EVs as non-invasive biomarkers in gynecological tumors.

**EV source**	**Isolation method**	**Type of molecule (protein/miRNA)**	**Cohort (n)**	**Clinical application**	**References**
Plasma	ExoGAG (Nasas Biotech)	ANXA2 and L1CAM	41 patients with EC vs. 20 healthy controls	Diagnosis/ prognosis	(48)
Urine	UC	miR-200c-3p	22 patients with EC vs. 5 symptomatic controls	Diagnosis/prognosis	(52)
Peritoneal lavage	UC	miR-383-5p, miR-10b-5p, miR-34c-3p, miR-449b-5p, miR-34c-5p, miR-200b-3p, miR-2110, and miR-34b-3p	25 patients with EC vs. 25 healthy controls	Diagnosis	(53)
Serum	miRCURY (Qiagen)	miR-93 and miR-205	100 patients with EC vs. 100 healthy controls	Diagnosis/prognosis	(55)
Plasma/serum	UC	TGF-beta and MAGE3/6	22 patients with OC vs. 10 patients with serous cysts vs. 10 healthy controls	Diagnosis	(74)
Plasma	UC	Claudin 4	63 patients with OC vs. 50 healthy controls	Diagnosis	(69)
Ascites	UC	FasL and TRAIL, TCR, CD20, HLA-DR, B7-2, HER2/neu, CA125 and histone H2A	35 patients with OC	Immune system regulation	(67)
Serum	UC	CLIC4, PK1, AIMP1, SNX3, protein FAM49B, FERMT3, TUBB3 and lactotransferrin	10 patients with OC vs. 10 healthy women	Diagnosis	(71)
Serum	Immune isolation and nano/optical detection ExoCounter	CD9/HER2	50 patients with OC vs. 63 healthy controls	Diagnosis	(72)
Plasma	Precipitation ExoEasy Maxi kit (Qiagen)	GSN, FGG, FGA and LBP	40 patients with OC vs. 40 healthy women	Diagnosis/prognosis/ therapeutic target	(73)
Ascites	UC	MMP1	48 patients with OC vs. 12 benign disease	Prognosis	(75)
Serum	EpCAM based immunoisolation	miR-21, miR-141, miR- 200a, miR-200c, miR-200b, miR-203, miR-205 and miR-214	50 patients with OC vs. 10 patients with adenomas vs. 10 healthy women	Diagnosis	(76)
Serum	Precipitation Total Exosome Isolation Reagent (Invitrogen)	miR-200b and miR-200c	163 patients with OC, 20 patients with benign ovarian diseases and 32 healthy women	Diagnosis/prognosis	(78)
Serum	Precipitation ExoQuick (System Bioscience)	miR-100	20 patients with OC and 20 healthy women	Diagnosis	(79)
Serum	Precipitation Total Exosome Isolation Reagent (Invitrogen)	miR-222-3p	6 patients with OC vs. 6 healthy controls	Diagnosis/therapeutic target	(77)
Serum	UC	miR-99a-5p	62 patients with OC vs. 26 patients with benign ovarian tumors vs. 20 healthy volunteers	Diagnosis/therapeutic target	(80)
Plasma	UC	Up: miR-106a-5p, hsa-let-7d-5p, and miR-93-5p Down: miR-185-5p, miR-122-5p, and miR-99b-5p	30 patients with OC vs. 30 healthy volunteers	Diagnosis	(81)
Plasma	Precipitation ExoQuick (SystemBioscience)	Up: miR-21, miR-100, miR-200b, and miR-320 Down: miR-16, miR-93, miR-126, and miR-223	106 patients with OC vs. 8 patients with ovarian cystadenoma vs. 29 healthy women	Diagnosis /prognosis	(82)
Plasma	Precipitation ExoQuick (System Bioscience)	miR-181a, miR-1908, miR-21, miR-486 and miR-223	30 patients with OC (15 platinum resistant vs. 15 platinum sensible)	Therapy prediction	(83)
Pleural and peritoneal effusions	Precipitation ExoQuick (System Bioscience)	miRNAs 21, miRNA23b and 29a	86 patients with OC	Prognosis	(84)
Urine	UC	miR-30a-5p	39 patients with OC vs. 26 patients with benign gynecological disease vs. 30 healthy controls vs. 40 patients with gastric/colon cancer	Diagnosis/ therapeutic target	(85)

*UC, ultracentrifugation*.

Other challenging line of work includes validation of EVs for drug delivery (86). This possibility is supported by evidence of the tissue tropism of EVs, mediated by surface molecules that might eventually be translated to specific tumor targeting and subsequent drug delivery. Liposomes are the most illustrative example of versatile clinically available drug delivery vehicles (87). Both the lipid membrane and interior space are tunable for loading of hydrophobic and hydrophilic drugs, respectively. Likewise, functionalization of EVs is a promising alternative for the development of diagnostic tests predicting organ-specific metastasis and for more efficient therapies impairing metastasis. For example, exosomal integrins have been shown to direct organ-specific colonization by fusing with target cells in a tissue-specific fashion (88). Efficient functionalization of EVs, control of the yield and stability of the therapeutic cargo, purification and production scaling methods, sustained delivery during extended periods compatible with clinical timings, and appropriate and specific preclinical study designs are major challenges in the translation of these technologies into clinical practice for many tumor types, including gynecologic tumors.

For effective translation of EV-based analysis into clinical practice, substantial scientific effort will be required, involving both basic-science researchers and clinicians. In addition, this work should follow the recommendations of the International Society for Extracellular Vesicles (ISEV) and various working groups supporting the harmonization of protocols to improve the reproducibility of procedures and kits for EV analyses (89, 90), and to address relevant clinical questions in adequate clinical cohorts.

## Conclusions

Herein, we have reviewed the potential of cEVs in the development of gynecologic cancer biomarkers and therapies. Liquid biopsy approaches are minimally invasive and provide a comprehensive picture of tumors, thus providing a new opportunity for the application of personalized oncology. In particular, cEVs present in blood, ascites, or urine have shown great potential as biomarkers in the clinical management of gynecologic tumors. Both protein and miRNA EV cargo have shown promise in preclinical studies as biomarkers for early detection, prognosis, and prediction of the response to therapy and the acquisition of resistances in endometrial or ovarian tumors. However, this field is in its infancy, and many challenges must be met before clinical utilization of EVs can be achieved for detection, monitoring, and therapy selection for gynecologic tumors.

## Author Contributions

All authors have contributed to the bibliographic review and manuscript preparation.

## Conflict of Interest

MA has ownership in Nasasbiotech. This company commercializes ExoGAG. CH was employed by Nasasbiotech, S.L. The remaining author declares that the research was conducted in the absence of any commercial or financial relationships that could be construed as a potential conflict of interest.
